# Experimental Models of Weight Cycling: A Narrative Review of Protocol Design and Metabolic Outcomes

**DOI:** 10.1155/jobe/2128461

**Published:** 2026-08-08

**Authors:** Isabela Macedo Lopes Vasques-Monteiro, Carolline Santos Miranda, Daniela de Barros Mucci, Bianca de Oliveira Saguie, Rafael Loureiro Simões, Fabiane Ferreira Martins, Vanessa Souza-Mello, Julio Beltrame Daleprane

**Affiliations:** ^1^ Department of Basic and Experimental Nutrition, Laboratory for Studies of Interactions Between Nutrition and Genetics, LEING, Rio de Janeiro State University, Rio de Janeiro, Brazil, uerj.br; ^2^ Laboratory of Molecular and Cellular Pharmacology, Roberto Alcântara Gomes Biology Institute, State University of Rio de Janeiro, Rio de Janeiro, Brazil, uerj.br; ^3^ Department of Morphology, Federal University of Rio Grande do Norte, Natal, Rio Grande do Norte, Brazil, ufrn.br; ^4^ Laboratory of Morphometry, Metabolism and Cardiovascular Diseases, Biomedical Center, Institute of Biology, Rio de Janeiro State University, Rio de Janeiro, Brazil, uerj.br

**Keywords:** experimental protocols, metabolic dysfunction, obesogenic memory, preclinical model, sex difference, yo-yo effect

## Abstract

Weight cycling (WC), characterized by repeated weight loss and regain, has been associated with adverse health effects, though evidence remains inconsistent. The aim of this review is evaluating WC protocols used in rodent studies, with a particular focus on how variations in experimental design and cycle periodization influence metabolic outcomes. All included studies were conducted in C57BL/6 mice, with protocols alternating between normocaloric and hypercaloric diets, in a variety of protocols, mainly in the periodization of cycles, diet composition, and sex of the animals. Despite evidence suggesting greater resistance to metabolic disorders associated with WC, studies in females are still scarce. Frequent cycling was associated with worsened metabolic outcomes, whereas prolonged weight‐loss phases did not reverse obesity‐induced alterations. Common findings included increased feed efficiency, loss of lean mass, persistent insulin resistance, and impaired glucose tolerance, alongside adipose tissue remodeling and low‐grade inflammation. Divergent results appeared in relation to inflammation and lipid deposition in peripheral tissues. Collectively, the need for more standardized, sex‐inclusive experimental designs will improve mechanistic insight and translational relevance.

## 1. Introduction

Obesity affects approximately 16% of the global adult population, with projections exceeding one billion affected individuals by 2030 [1, 2]. Weight loss is the primary therapeutic strategy and improves obesity‐related metabolic risk factors [3]. However, long‐term weight loss maintenance remains a major challenge. Most individuals regain lost weight within 1 year, and even modest weight regain (2%–6%) is sufficient to attenuate or reverse the metabolic benefits achieved with clinically significant weight loss [4–7]. This highlights the critical need for strategies that promote not only effective weight loss but also long‐term weight maintenance.

Data suggest that weight loss and weight maintenance are physiologically and psychologically different in many respects. Therefore, individuals may require distinct interventions aimed at temporarily sustaining a negative energy balance during weight loss versus permanently maintaining energy balance after weight loss [8]. However, weight regain occurs regardless of how weight loss is achieved, whether through lifestyle changes, including behavioral therapy, bariatric surgery, or pharmacotherapy [9].

The process of periodically losing and gaining weight, similar to the up‐and‐down movement of a yo‐yo, is commonly called “weight cycling,” “weight fluctuation,” or the “yo‐yo effect,” and presents its own set of health challenges [10]. Although the potential health risks associated with weight cycling (WC) are concerning, it is equally important to recognize the well‐documented cardiometabolic risks of sustained obesity, including chronic inflammation, insulin resistance, and cardiovascular complications [11]. This underscores the need for personalized interventions that balance the potential harms of WC with the adverse effects of persistent obesity.

However, the evidence remains conflicting. While some studies suggest potential risks, others conducted in human populations have found no significant association between WC and an increased risk of metabolic diseases when compared to individuals with sustained overweight or obesity [12–14]. These discrepancies may stem from differences in study design, population characteristics, and the methods used to define and assess WC [15]. Additional factors, such as the magnitude of weight fluctuations, the frequency of cycles, and individual physiological responses, likely contribute to the variability in outcomes [16].

Weight regain is predominantly based on associative data, highlighting the need for research into the causal mechanisms behind these observed patterns [17]. In this context, research on WC in rodent models emerged in the 1990s to address metabolic issues and methodological limitations inherent to studies in humans [18]. However, even among rodent studies, conflicting findings have been reported [19–21].

This inconsistency largely arises from substantial heterogeneity in experimental design, including differences in diet composition, duration of high‐fat feeding, length of weight‐loss phases, number of cycles, and sex distribution. As a result, it remains unclear which metabolic alterations are robust consequences of WC per se and which are driven by protocol‐specific factors.

Accordingly, the aim of this review is evaluating WC protocols used in rodent studies, with a particular focus on how variations in experimental design and cycle periodization influence metabolic outcomes. This review does not aim to estimate pooled effects of WC but rather to critically examine how experimental design choices influence reported metabolic outcomes in preclinical models.

## 2. Experimental Protocols Used in Rodent Models to Simulate Body Mass Cycling

This review was designed as a narrative, methodology‐oriented synthesis. Accordingly, formal protocol registration, PRISMA reporting, and quantitative risk‐of‐bias assessment tools were not applied. The focus of this review was to identify and compare experimental designs used in rodent WC studies, with the aim of highlighting sources of methodological heterogeneity rather than estimating pooled effects.

Studies were identified through searches in PubMed and restricted to articles published within the last 7 years. This time frame was intentionally selected to capture contemporary WC protocols incorporating modern obesogenic diets, immunometabolic endpoints, and sex‐specific analyses, while avoiding redundancy with earlier reviews that have already covered foundational literature. Only studies that clearly reported the periodization of the protocols and the type of diet of WC were selected.

**FIGURE 1:**
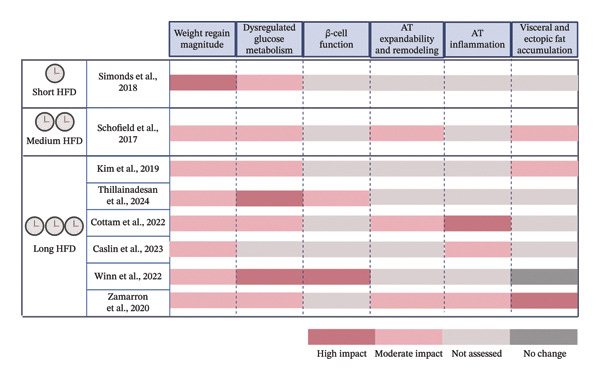
Protocol‐dependent metabolic impact of weight cycling. Integrative overview summarizing the metabolic impact of different weight cycling protocols across experimental models. Color intensity indicates the magnitude of metabolic impact. Short‐, medium‐, and long‐term HFD correspond to 2, 6, and ≥ 8 weeks of high‐fat diet exposure, respectively. The main results are compared with the control groups. This figure represents a qualitative synthesis of published findings. Abbreviations: AT: adipose tissue; HFD: high‐fat diet. Created in https://BioRender.com.

Table 1 summarizes the main protocol characteristics, highlighting variability in diet composition, duration of high‐fat exposure, and restriction phases. To facilitate a more integrated interpretation, Figure 1 synthesizes these protocols and their associated metabolic outcomes, illustrating how distinct WC paradigms lead to divergent responses when compared with control conditions. The main results are compared with the control groups.

**TABLE 1:** Experimental weight cycling (WC) protocols and main results.

Reference	Strain/age	Sex	HF phase (weeks)	Restriction phase (weeks)	Cycles	HF diet (% fat)	Restriction diet (% fat)	Biological sample	Main results
Simonds et al. [22]	C57BL/6J, 4 weeks	M	2	2	8	43%	12%	Plasma	Energy expenditure (↓↓) Glucose tolerance (↓) Appetite for high‐fat foods (↑↑) Basal plasma insulin (↔) Insulin sensitivity (↔)

Schofield et al. [23]	C57BL/6, 8–10 weeks	M	6	6	3	60%	10%	WAT, brain, and plasma	Visceral fat deposition (↑) Plasma fasting glucose (↑) Plasma fasting insulin (↑) Leptin level (↑) Resistin level (↑) *Mc4r*, *leptin receptor,* and *dopamine* in the hypothalamic or ventral tegmental area (↔)

Kim et al. [20]	C57BL/6N, 4 weeks	M	8 (1^st^ cycle) and 4 (3^rd^ cycle)	4	3	60%	4%–7%	WAT, liver and serum	Gluconeogenesis (↑) Fasting blood glucose (↑) *Tnf-α* in liver (↑) *Tgf-β1* in liver (↑)

Thillainadesan et al. 2024 [21]	C57BL/6J, 12 weeks	M	8	8	3	45%	12%	Islet ex vivo	Fasting insulin (↑) Insulinemia (↑) Glucose tolerance (↓) Peripheral insulin sensitivity (↓) Pancreatic beta cell function (↓)

Cottam et al. [24]	C57BL/6J, 8 weeks	M + F	9	9	3	60%	10%	WAT (vascular fraction)	Glucose tolerance (↓) AT T‐cell population (↑) Type 2 regulatory cells (↓) Antigen‐presenting cells (↑) T cell (↓) Lipid handling in macrophages (↑)

Caslin et al. [25]	C57BL/6J, 8 weeks	M + F	9	9	3	60%	10%	WAT (vascular fraction) and in vitro models using bone marrow	Innate immune response (↑) Metabolism of AT macrophages (↑) Basal secretion of TNF‐α (↑)

Winn et al. [26]	C57BL/6J, 8 weeks	M	9	9	3	60%	10%	Pancreatic islets and liver	Hepatic glycogen (↔) Hepatic triglycerides (↓) Insulin secretory capacity (↓) *Mafa, Pdx1, Nkx6.1, Ucn3* in pancreatic islets (↓) Pancreatic beta cell function (↓) Insulin in pancreatic islets (↓)

Zamarron et al. [27]	C57BL/6J, 6 weeks	M	12 (1^st^ cycle) 6 (3^rd^ cycle)	8	3	60%	13.4%	WAT (vascular fraction) and liver	Liver mass (↑) Hepatic triglyceride (↑) Serum transaminase (↑) Insulin concentrations (↑) CD11c^+^ AT macrophages, CD8^+^ AT T cells, and Col1^+^ preadipocytes (↑) Proinflammatory cytokines (↑)

Zamarron et al. [27]	C57BL/6J, 6 weeks	M	12 (1^st^ cycle) 6 (3^rd^ cycle)	24	3	60%	13.4%	WAT and liver	eWAT expandability (↑↑) Ectopic accumulation of lipids in the liver (↑)

Considering the studies in Table 1, those that analyzed weight regain through periodization in detailed weekly intervals were selected. It is well known that there is significant methodological variability in the reproduction of WC in animal studies; despite this, most studies indicate that WC induces unique metabolic effects, some of which are detrimental [20–27]. However, divergent findings persist [20, 21], likely due to differences in experimental protocols used.

Frequent periods of WC have been shown to result in decreased weight loss during subsequent energy restriction phases, accompanied by increased weight gain and feed efficiency with each new cycle of high‐energy diet [22]. Feed efficiency reflects the efficiency with which consumed calories are converted into body mass and is frequently used to assess metabolic adaptations in obesity and weight‐cycling models [28]. During re‐exposure to calorie‐restricted diets, animals exhibited a reduction in caloric intake of approximately 7 kcal per day [22]. This reduced food intake, coupled with attenuated weight loss, suggests that animals adapt to future caloric restriction by reducing their energy expenditure. Interscapular brown adipose tissue (iBAT) temperature in mice subjected to multiple cycles is lower than in weight‐matched mice maintained on a high‐calorie diet [22]. The reduction in energy expenditure after repeated WC may be related to a loss of brown adipose tissue (BAT); however, further studies are needed to confirm this association [10].

The duration of initial exposure to a high‐calorie diet varies between studies, with significant differences in outcomes depending on the rodent strain and diet composition. Certain strains of rats and mice are highly susceptible to diet‐induced obesity (DIO) when consuming diets where 40%–60% of energy is derived from fat for a period of 8–12 weeks [29]. Among these, the C57BL/6 mouse strain, which is commonly used in preclinical studies (as summarized in Table 1), shows a rapid response to DIO, developing glucose intolerance within just 3 days of beginning a high‐fat diet (HFD, 45% fat) and significant obesity after just 5 weeks [30]. Recently, preclinical models have shifted to incorporate diets high in lipids and sugars, often referred to as “Westernized diets,” to better replicate human dietary patterns. These diets are metabolically more damaging than traditional HFDs, exacerbating metabolic disorders and inflammation [29]. In the context of WC studies, evidence indicates that the inflammatory potential of the diet during the weight maintenance phase is a stronger predictor of weight regain than the macronutrient composition of the diet. This suggests that inflammation plays a critical role in the physiological processes that drive WC and subsequent metabolic dysregulation [31].

In one study, a 2‐week interval was chosen to promote significant weight loss, allowing multiple WCs before the animals reached the aging phase. Dietary cycling began after 10 weeks on a HFD to ensure the development of obesity before initiating the cycling protocol [22]. Shorter intervals, such as 4 weeks or less, are considered insufficient for animals to achieve the expected weight on a high‐calorie diet. On the other hand, a 6‐week cycle provides adequate time for stabilization of body mass and food intake according to the prescribed diet, effectively reducing confounding factors related to body weight changes [23]. These variations in cycle length highlight the importance of protocol optimization to achieve consistent and interpretable results in WC studies.

In the 9‐week periodization model, introduced in 2013, the authors proposed an initial 9‐week HFD phase to induce obesity and immune cell infiltration in AT, specifically involving CD4+ Th1, Th2, and CD8+ T cells [24]. The subsequent 9 weeks of a low‐fat diet were intended to promote weight loss, while the final 9‐week re‐exposure to a HFD was intended to induce obesity once again [32]. Using this model, researchers have characterized a subset of immune cell populations in rodents subjected to this WC. Furthermore, it was found that weight regain leads to the accumulation of T cells in AT, which negatively affects systemic glucose tolerance. Additionally, weight regain may induce innate immunological memory in macrophages within AT and sustain an inflammatory phenotype in immune cells [24–26].

Even prolonged weight loss (6 months), followed by shorter re‐exposure to a HFD (6 weeks), fails to restore epididymal white adipose tissue (eWAT) expansion capacity [27]. CD11c+ macrophages and crown‐like structures persist in AT despite prolonged weight loss, which is also associated with persistent insulin intolerance [33]. Given this prolonged persistence, Zamarron et al. suggest that a single exposure to obesity may have a lasting impact on eWAT function and architecture. The long‐term consequences of obesity, even after weight loss, may impair metabolic flexibility [27].

Large fluctuations in body weight result in a mismatch between blood glucose and insulin secretion in mice. Short and frequent periodization of WC found no impact on insulin values [22], but a longer periodization found significantly elevated fasting insulin levels [21]. Data from a mouse study suggest that there is a loss of adequate β‐cell compensation during the longer periodized cycle (from 6 weeks onwards) that is normally observed only during obesity. Pancreatic plasticity is thought to be challenged by large variations in body weight due to reduced expression of transcription factors essential for β‐cell function (*Mafa, Pdx1, Nkx6.1,* and *Ucn3*) and lower islet insulin content [26].

Despite the variability of experimental protocols, evidence suggests that three dietary transitions (a high‐calorie diet followed by a lower‐calorie diet and then re‐exposure to a high‐calorie diet) are sufficient to model WC in rodents. More frequent cycling correlates with worsening metabolic outcomes, characterized by greater weight gain with each new high‐calorie cycle and greater difficulty losing weight during subsequent low‐calorie cycles, although no effects on basal insulin or insulin sensitivity were observed [22]. A prolonged weight loss period (lasting 6 months) still seems insufficient to mitigate the damage caused by the initial high‐calorie diet [27].

It is well established that female rodents are less susceptible to DIO compared to males [24]. Sex differences were observed during the recovery of body weight, fat mass, and lean mass. After weight regain, male mice did not achieve body mass values like their continuously obese counterparts, whereas females had significantly greater body weight, lean mass, and relative fat mass after WC. The authors attribute this partly to females’ lower relative body mass [34].

Sex differences in body weight regain are strongly influenced by estrogen signaling. Estrogen, acting primarily through estrogen receptor alpha (ERα), plays a central role in maintaining metabolic flexibility by regulating adipose tissue (AT) distribution, enhancing insulin sensitivity, and limiting AT inflammation. ERα activation promotes preferential subcutaneous fat storage, suppresses ectopic lipid accumulation, and improves mitochondrial function in adipocytes and skeletal muscle [35].

In parallel, estrogen signaling modulates adiponectin, which exerts insulin‐sensitizing and anti‐inflammatory effects. Higher adiponectin levels in females contribute to improved glucose homeostasis and protection against metabolic deterioration during inflammation [36, 37]. This protective axis is disrupted in estrogen‐deficient models, as demonstrated in ovariectomized (OVX) or aged female rodents, which exhibit reduced adiponectin levels, increased visceral adiposity, impaired glucose tolerance, and heightened inflammatory responses following HFD exposure [38]. OVX animals exhibit rapid weight gain following surgery due to alterations in energy intake, energy expenditure, or both, with total body weight significantly exceeding pre‐OVX [39].

Corroborating this, a study investigating the yo‐yo diet found that the negative effect of WC was more pronounced on the intestinal morphology of male mice than in females [40]. These females showed greater morphological preservation of the colon, maintenance of goblet cell integrity, and epithelial structure at the end of WC [40]. Understanding these differences is essential to develop personalized interventions and increase the translational relevance of preclinical WC research. Investigations into the consequences of WC in females are limited.

It is evident that the ideal periodization is one that allows sufficient exposure time for the animal to metabolically respond to the type of dietary insult it is receiving. For example, during exposure to a high‐calorie diet, the transition to another phase of the cycle should only occur after the animals present the expected metabolic responses, such as increased body mass and associated metabolic changes. Above all, the methodological choice for WC studies in rodents depends on the specific metabolic goal, available time, financial resources, and experimental facilities.

## 3. Metabolic Impact of Weight Cycling in Rodents

Weight regain after weight loss has been associated with the development of metabolic abnormalities, particularly in AT, liver, gut, and central nervous system (CNS). Animals subjected to WC often exhibit distinct metabolic profiles from those continuously fed an obesogenic diet throughout the experiment. These differences, which will be explored in this section, emphasize the unique physiological impacts of WC.

### 3.1. Adipose Tissue Remodeling

AT plays a vital role in energy balance and therefore in the genesis of obesity [41]. Adipocytes are the only cells that have specialization to store lipids without compromising their functional integrity. At least three metabolically distinct types of adipocytes are found in humans: white, beige, and brown adipocytes [42]. Unlike white adipocytes, which store extra energy as triacylglycerol (TAG), classic beige and brown adipocytes are distinguished by their unique ability to dissipate mitochondrial energy as heat via uncoupling protein 1 (UCP1) [43, 44].

AT is the most studied tissue in weight loss and regain research [20, 21, 23, 27]. Weight loss is accompanied by loss of fat mass, resulting in adipocyte shrinkage without concomitant remodeling of the extracellular matrix (ECM). This mismatch is frequently associated with mechanical stress in both the matrix and the adipocytes, impairing lipolysis and hindering the release of fatty acids from the adipocytes [45]. Interestingly, research indicates that adipocyte replacement may play a role in mitigating this mechanical stress in humans. However, this adaptive response is intricately linked to weight regain, since the restoration of adipocyte volume and number is often accompanied by an increase in body weight [46, 47]. It is not surprising that the pronounced obesogenic effect of WC is increased adipocyte recruitment and hypertrophy [15, 48].

Matrix metalloproteinases (MMPs) play a fundamental role in ECM remodeling, and their deregulation has been described in human obesity and in experimental studies [49]. During weight loss, intense MMP activity has been reported, and the inflammatory profile remains in AT. Inhibition of MMPs during weight gain interferes with ECM remodeling, leading to reduced adipocyte size in subcutaneous AT and inflammatory relief with increased adiponectin and IL‐10 in white adipose tissue (WAT) [50]. Since then, MMP inhibitors have emerged as a potential target of interest to mitigate inflammation during WC [50].

Due to its endocrine function, AT is also responsible for producing circulating adipokines involved in metabolism [51]. WC may decrease the production of adiponectin, a potent anti‐inflammatory effector, while increasing the expression of resistin, an inflammatory signaling molecule that contributes to IR. This suggests that metabolic disturbances linked to WC may be associated with adipokine dysregulation [52, 53]. One of the latest adipokines to be discovered, CTRP3, is associated with improved IR by reducing inflammation and insulin signaling transduction [54]. In the context of WC, CTRP3 expression decreases in AT concomitantly with decreased PKB phosphorylation (Ser473), PI3K protein, and *Glut4* gene expression, resulting in activation of metabolic pathways that contribute to decreased insulin efficacy [55].

There is also an alteration in the expression of clock genes, with reduced expression of *Dbp* and *Tef* and a tendency toward increased expression of *Arntl/Bmal1*, which are involved in the regulation of the circadian cycle, probably an adaptive response to repeated fasting and refeeding cycles [15]. These findings provide insights into the impact of an obesogenic diet on AT functionality and architecture. On the other hand, metabolic and molecular studies regarding WC in iBAT remain scarce.

Furthermore, a study investigated the cell types, molecular events, and regulatory factors that remodel the human AT and, therefore, metabolic health, in obesity and therapeutic weight loss. It was observed that senescence is potently reversed by weight loss. Weight loss reduces adipocyte hypertrophy and biomechanical restriction pathways, activating overall metabolic flux and bioenergetic substrate cycles that may mediate systemic improvements in metabolic health [56].

### 3.2. Obesogenic Memory

The immunological characteristics of AT have become crucial in understanding the ease of weight regain. Adipocytes, T cells, and macrophages in AT have been presented less inflamed after weight loss but do not return to the levels observed in mice never exposed to obesity [57]. This persistent metabolic inflammatory state is referred to in the literature as “Trained immunity” [58, 59].

More recent evidence has demonstrated that innate immune cells are capable of developing long‐lasting functional memory mediated by epigenetic reprogramming, occurring at the level of histone H3 methylation, which promotes durable changes in chromatin accessibility and gene expression [60–62]. Trained immunity is sustained by epigenetic and metabolic reprogramming of myeloid cells and is characterized by activation of the Akt–mTOR–HIF‐1α pathway. In human monocytes, β‐glucan training induces histone modifications associated with transcriptional activation and increased expression of glucose metabolism–related genes, promoting enhanced glucose uptake, lactate production, and an increased NAD^+^/NADH ratio, indicative of a persistent metabolic shift toward aerobic glycolysis [63].

Inhibition of Akt, mTOR, or HIF‐1α blocks the induction of trained immunity, and myeloid‐specific HIF‐1α deficiency prevents its development in vivo, demonstrating that this pathway constitutes the central metabolic basis of trained immunity [64]. In parallel, epigenetic alterations are also associated with the concept of “obesogenic memory” or “metabolic memory.” In retinal capillary endothelial cells, hyperglycemic conditions reduce the expression and activity of sirtuin 1 (SIRT1), an effect that persists after glycemic normalization due to the activation of histone acetyltransferases (HATs), which promote lysine residue acetylation on histones at the promoter regions of proinflammatory genes [65].

SIRT1 inhibition results in increased acetylation of targets such as Foxo1 and the p65 subunit of NF‐κB, culminating in oxidative stress and sustained inflammatory responses even after glycemic restoration [61, 66]. Epigenetic modifications play a central role in adipocyte identity and emerge as key mechanisms underlying obesogenic memory in AT [62, 67, 68].

Obesity induces durable changes in chromatin accessibility and transcriptional regulation that persist even after weight loss, resulting in sustained activation of inflammatory and ECM remodeling pathways concomitant with repression of adipocyte‐specific metabolic pathways [69]. This stable cellular memory is reinforced by functional alterations, such as increased glucose uptake in adipocytes and impaired adipogenic capacity of the stromal vascular fraction, suggesting that metabolic alterations persistently modulate the molecular and functional responses of AT [62].

AT macrophages exhibit persistent molecular alterations that sustain obesogenic memory. During obesity, tissue‐resident macrophages maintain expression of Mrc1 (CD206) but display a durable loss of Cd163 expression, indicating transcriptional reprogramming of the M2 phenotype that persists for weeks after weight loss [24]. Concurrently, both macrophage subpopulations acquire a more proinflammatory profile, along with stable changes in population composition, including expansion of lipid‐associated and nonperivascular macrophages in epididymal AT [24]. Persistent elevation of F4/80 expression and maintenance of low‐grade inflammation after weight loss further support the existence of innate memory in adipose macrophages, which contributes to weight regain and challenges in obesity treatment [70].

Global transcriptomic analysis demonstrates that weight regain activates adaptive immune responses in WAT of mice [71]. Consistently, a significant increase in CD4^+^ effector T lymphocytes, including Th1 and Th17 subpopulations, has been observed in epididymal AT of mice with a history of obesity. Chronic inflammation and immune cell infiltration in AT are associated with adipokine secretion and accumulation of CD4^+^ and CD8^+^ effector memory T cells, which impair insulin sensitivity in WC models [62, 72].

Together, these findings indicate that WAT inflammation persists in obesity and that immune cells, particularly adaptive immune cells, constitute a central component of the mechanisms associated with weight regain. In addition, further studies are required to investigate inflammatory cytokine production in the plasma of animals subjected to WC.

### 3.3. Systemic Metabolic Repercussions

Overall, WC was frequently associated with exacerbated weight gain and increased food efficiency, defined as the weight gained relative to energy consumed. This phenomenon is accompanied by increased fat accumulation and significant changes in body composition, including a reduction in lean mass and physical strength. These changes may not only amplify metabolic dysfunction but also contribute to long‐term challenges in maintaining overall metabolic health, highlighting the complex consequences of WC [11, 15]. These systemic changes may contribute to persistent insulin resistance, impaired glucose metabolism, reduced adiponectin levels, and an altered immune cell profile [15, 57, 68, 73].

The impact of WC on the liver remains controversial. Inia et al. reported that animals subjected to WC showed a trend toward increased microvesicular steatosis but also exhibited reduced hepatic inflammation, with fewer inflammatory cell aggregates and F4/80‐positive crown‐like structures compared to those on a continuous HFD and no evidence of hepatic fibrosis [48]. Moreover, prolonged weight loss was found to reduce hepatic proinflammatory gene expression, in contrast to visceral AT [74]. Conversely, Kim et al. showed that WC led to increased hepatic expression of TNF‐α and TGF‐β1 [20].

The interactions between the gut microbiota and weight regain have been poorly explored. Although specific mechanisms remain unclear, microbial strains such as *Bifidobacterium* in the gastrointestinal tract and eosinophilic *Bifidobacterium* in the mucosa are inversely associated with weight loss [75]. When calorie restriction was analyzed, the presence of Christensenellaceae has been identified as a significant biomarker for reduced weight regain [76]. Caloric restriction has been reported to increase the surface area of the jejunal villi, which, as demonstrated in Zhang’s study, may significantly contribute to weight regain by increasing intestinal absorption [77].

Neuronal inflammation in the CNS, particularly in the hypothalamus, is associated with weight regain [72]. The hypothalamus and hindbrain play a pivotal role in the homeostatic regulation of food intake and energy balance in response to peripheral and environmental signals. WC was associated with substantial glucose dysregulation, endocrine adaptations, and changes in metabolic rate, possibly linked to changes in the secretion of circulating hormones such as orexigenic ghrelin, anorexigenic leptin, insulin, GLP‐1, CKK, and PYY [68]. These endocrine changes are thought to lead to hyperphagia and promote weight regain. In humans, these hormonal changes appear to persist for at least a year after weight loss [78].

An analysis of the “biological sample” column in Table 1 reveals that most studies focus on central tissues implicated in obesity, such as WAT, pancreas, and liver. However, adjacent tissues that are less frequently studied in the context of WC may offer critical insights into unresolved questions about metabolism after weight regain. These tissues include BAT, the hypothalamus, and the intestine, which play significant roles in energy balance and metabolic regulation.

**FIGURE 2:**
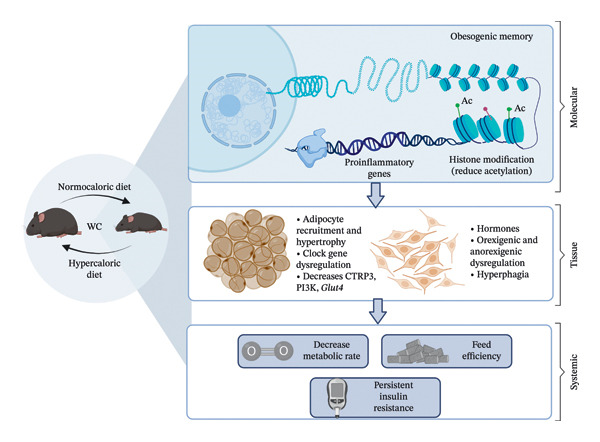
Metabolic impacts of weight cycling in rodents. Epigenetic changes established during obesity may persist after weight loss, characterizing obesogenic memory, which is believed to contribute to weight regain. Common findings across experimental protocols are highlighted in the figure. The results presented reflect metabolic alterations uniquely associated with weight cycling. Abbreviations: Ac: acetylation; WC: weight cycle. Created in https://BioRender.com.

Future research should focus on investigating the inflammatory, metabolic, and structural repercussions in these underexplored tissues, along with examining the composition and functional dynamics of the gut microbiota. Such studies are essential for elucidating the mechanisms driving the physiological and metabolic impacts of WC. Figure 2 provides an overview of the metabolic effects of WC observed in rodent models, highlighting molecular, tissue, and systemic involvement.

## 4. Intervention Strategies and Future Perspectives

Weight regain affects approximately 80% of individuals who intentionally lose more than 10% of their body weight, with weight regain occurring within a year [6]. Therefore, intervention strategies should be studied to prevent weight regain and/or minimize its metabolic impacts. Resistant starch (RS) supplementation during WC has been shown to reduce damage to the gastrointestinal mucosa, with a decrease in the density of inflammatory cells in the small intestine of male mice [40]. RS is a type of dietary fiber fermented by the gut microbiota, leading to the production of short‐chain fatty acids [79]. Type 4 RS, a chemically modified starch that resists digestion in the small intestine and reaches the colon largely intact [80], is the most widely used due to its greater chemical and physical resistance, as well as its superior ability to reduce oxidative stress in DIO compared to other RS subtypes [81].

Fructooligosaccharide (FOS) supplementation, another type of fiber, in a HFD preserved the subset of RORγt+ CD4 T cells, pTregs, and Th17 cells. Additionally, FOS supplementation requires type 2 conventional CD11b+ dendritic cells (cDC2) to maintain intestinal Th17 cells. This type of dendritic cell is essential for regulating the immunological and metabolic effects of FOS [82]. Given this context, it is estimated that the immunological effect of fibers may alleviate the metabolic damage caused by WC, but more in‐depth studies are needed to confirm this hypothesis.

The use of anti‐obesity drugs (AOMs) is underutilized after bariatric surgery to prevent weight regain. A multicenter study found that only the use of topiramate was able to demonstrate a statistically significant response against weight regain, with patients being twice as likely to lose at least 10% of their weight when submitted to this medication [83]. More recently, liraglutide has been investigated in this context. Liraglutide at doses of 1.2 mg [84] to 3 mg/day [85] was associated with weight loss in individuals who were already experiencing weight regain after Roux‐en‐Y gastric bypass (RYGB). The use of this medication resulted in a 14.8% reduction in body mass index over 36 months, and 21% of individuals reached their lowest postsurgery weight within 14 months, proving to be an effective treatment for weight regain [84, 85]. It is also hypothesized that these same AOMs may have sufficient metabolic impacts in the context of WC to minimize weight regain. However, further studies are needed to confirm this hypothesis.

In recent years, incretin analogs have been widely prescribed for the treatment of obesity and diabetes due to their potent effects on reducing body weight and improving glucose homeostasis. However, many patients do not maintain incretin analog therapy and consequently regain body weight rapidly. One study demonstrated that treatment with incretin analogs restored most of the gene expression dysregulated by obesity in AT. This same study revealed the effects of incretin analog therapy and its discontinuation in AT, highlighting the importance of dietary composition during and after incretin analog therapy [86].

Intermittent fasting is a nutritional strategy that has gained prominence for promoting weight loss and improvements in cardiometabolic markers, showing comparable effects to other hypocaloric diets in numerous randomized controlled trials and recent meta‐analyses. A clinical trial demonstrated that the practice of intermittent fasting in individuals with type 2 diabetes resulted in weight reduction and other metabolic benefits, while also emphasizing the importance of continuous monitoring and long‐term evaluation of outcomes [87]. Nevertheless, long‐term evidence suggests that following the discontinuation of any weight loss intervention, the body tends to regain part or all of the lost weight, a process that involves physiological adaptations such as reductions in resting energy expenditure and hormonal changes that increase appetite and promote fat storage [88].

Strategies to prevent weight regain in the first year after weight loss appear to be crucial for long‐term weight maintenance. Data show a 50% reduction in the risk of weight regain in individuals who successfully maintain weight loss for 2 years [6]. Therefore, intervention strategies should be intensified during the first year of weight loss. Strict adherence to weight maintenance strategies is essential for 89% of those who successfully maintain their weight, including elevated levels of physical activity and nutritional interventions [68]. There is a significant gap in interventions focusing on physical activity and mental health during weight loss, maintenance, and regain. Studies that investigate comprehensive lifestyle changes in individuals undergoing these processes may find a higher success rate in maintaining lost weight. Although WC has unique metabolic characteristics, its metabolic effects are less detrimental than persistent obesity. Therefore, efforts to achieve weight loss should not be discouraged [13].

## 5. Conclusion

Exploring WC in rodent models offers valuable insights into the complex metabolic impacts and biological adaptations that occur during weight loss and weight regain cycles. The variability in WC research protocols, including differences in diet composition, cycle duration, and age of animals at experiment start, highlights the diversity of approaches used to simulate WC. This methodological heterogeneity poses challenges to standardization, but careful considerations, such as appropriate periodization of dietary regimens, can help ensure meaningful and reproducible results.

Because this is a narrative review, the findings should be interpreted as descriptive and hypothesis‐generating rather than definitive. Nevertheless, the consistent emergence of protocol‐dependent patterns highlights methodological variability as a central factor driving discordant results in the literature.

Despite these challenges, rodent studies consistently reveal that the more cycles of weight loss and regain, the greater the exacerbation and metabolic dysfunction, underscoring the significant “metabolic cost” of WC. Additionally, effects on reduced energy expenditure, impaired glucose tolerance, and persistent insulin resistance were among the most consistently reported outcomes across different protocols, sexes, and tissues. In contrast, alterations in hepatic inflammation, fibrosis, and ectopic lipid accumulation showed substantial variability and were highly dependent on experimental design, duration of exposure, and tissue‐specific analyses.

The pronounced heterogeneity among preclinical WC protocols substantially constrains their translational relevance. Diet composition, cycle duration, and sex inclusion vary widely across studies; WC elicits heterogeneous and sometimes conflicting metabolic responses. This variability complicates the interpretation of the biological effects of WC itself and limits the translational relevance of preclinical findings.

Future preclinical WC studies should prioritize greater methodological transparency and harmonization. Standardizing key design elements, such as diet composition, duration and frequency of weight loss–regain cycles, and inclusion of both sexes, would facilitate cross‐study comparisons and improve biological interpretability. Moreover, integrating tissue‐specific metabolic and immunological endpoints across consistent experimental frameworks may help distinguish core consequences of WC from protocol‐dependent effects, thereby enhancing the value of preclinical models.

## Author Contributions

Conceptualization, Isabela Macedo Lopes Vasques‐Monteiro and Julio Beltrame Daleprane; data curation, Isabela Macedo Lopes Vasques‐Monteiro and Carolline Santos Miranda; formal analysis, Isabela Macedo Lopes Vasques‐Monteiro; investigation, Isabela Macedo Lopes Vasques‐Monteiro; methodology, Isabela Macedo Lopes Vasques‐Monteiro, Vanessa Souza‐Mello, Julio Beltrame Daleprane; project administration, Julio Beltrame Daleprane; writing–original draft, Isabela Macedo Lopes Vasques‐Monteiro; writing–review and editing, Isabela Macedo Lopes Vasques‐Monteiro, Carolline Santos Miranda, Daniela de Barros Mucci, Rafael Loureiro Simões, Bianca de Oliveira Saguie, Fabiane Ferreira Martins, Vanessa Souza‐Mello Julio Beltrame Daleprane; visualization, Isabela Macedo Lopes Vasques‐Monteiro, Carolline Santos Miranda, Daniela de Barros Mucci, Bianca de Oliveira Saguie, FFB, Vanessa Souza‐Mello, Julio Beltrame Daleprane; supervision, Julio Beltrame Daleprane.

## Funding

This study was supported by the Fundação Carlos Chagas Filho de Amparo à Pesquisa do Estado do Rio de Janeiro‐FAPERJ E‐26/211.193/2021; E‐26/201.234/2022; E‐26/210.332/2022 granted to Dr. Julio Daleprane; Coordenação de Aperfeiçoamento de Pessoal de Nível Superior–Brasil (CAPES), Finance Code 001 CAPES; Conselho Nacional de Desenvolvimento Científico e Tecnológico (CNPq).

## Disclosure

All authors have read and agreed to the published version of the manuscript.

## Conflicts of Interest

The authors declare no conflicts of interest.

## Data Availability

The data that support the findings of this study are available from the corresponding author upon reasonable request.
